# Exosomal MicroRNAs as Novel Cell-Free Therapeutics in Tissue Engineering and Regenerative Medicine

**DOI:** 10.3390/biomedicines10102485

**Published:** 2022-10-05

**Authors:** Eric Z. Zeng, Isabelle Chen, Xingchi Chen, Xuegang Yuan

**Affiliations:** 1 Department of Chemical and Biomedical Engineering, FAMU-FSU College of Engineering, Florida State University, Tallahassee, FL 32310, USA; 2 Los Altos High School, Los Altos, CA 94022, USA; 3 Department of Pathology & Laboratory Medicine, David Geffen School of Medicine, University of California-Los Angeles (UCLA), Los Angeles, CA 95616, USA

**Keywords:** extracellular vesicles, microRNA, biogenesis, cargo sorting, tissue repair and regeneration

## Abstract

Extracellular vesicles (EVs) are membrane-bound vesicles (50–1000 nm) that can be secreted by all cell types. Microvesicles and exosomes are the major subsets of EVs that exhibit the cell–cell communications and pathological functions of human tissues, and their therapeutic potentials. To further understand and engineer EVs for cell-free therapy, current developments in EV biogenesis and secretion pathways are discussed to illustrate the remaining gaps in EV biology. Specifically, microRNAs (miRs), as a major EV cargo that exert promising therapeutic results, are discussed in the context of biological origins, sorting and packing, and preclinical applications in disease progression and treatments. Moreover, advanced detection and engineering strategies for exosomal miRs are also reviewed. This article provides sufficient information and knowledge for the future design of EVs with specific miRs or protein cargos in tissue repair and regeneration.

## 1. Introduction

In general, all types of cells are able to secrete membrane-bound vesicles both in vivo and in vitro, which are broadly termed as extracellular vesicles (EVs). Initially, researchers found these lipid bilayer-enclosed secreted vesicles circulating across mammalian tissues/fluids and identified them as cellular debris or platelet dust [1]. Early studies of EV functions demonstrated their ability to remove cellular waste and lyse cellular compartments. Nowadays, intercellular communication is acknowledged as a major function of EVs. Due to the nature of “cell-secretion”, heterogeneity is a critical characteristic of EVs. Based on isolation and size characterization, EVs can be broadly (and roughly) classified as apoptotic bodies (ApoBs, ~500–5000 nm), microvesicles (MVs, ~100–1000 nm), and exosomes (~40–150 nm). ApoBs are distinctive populations originating from dying cells as a hallmark of apoptosis. At the final stage of apoptosis, cells disassemble into an abundance of ApoBs containing cellular fragments, which are precisely phagocytosed by macrophages, parenchymal cells, or neoplastic cells for degradation [2]. Little is known about ApoBs as therapeutic agents despite the fact that no inflammation or cytotoxicity is established by ApoBs. The major focus of ApoBs research concerns drug delivery vessels or diagnostics, as they carry a large number of proteins, lipids, RNA, and DNA molecules [3]. In this review, we mainly focus on MVs and exosomes, as an increasing body of evidence demonstrates their potential in disease diagnosis and therapeutic design. Specifically, exosomal micro-RNAs (exo-miRs), as one of the major EV cargos, are discussed in detail through EV biogenesis, secretion, and cargo sorting and packaging. Moreover, advanced studies on diagnostics, therapeutic applications, and bioengineering strategies of exo-miRNAs are also reviewed to provide insights for the future design of cell-free therapies with EVs and exo-miRs.

## 2. EV Biogenesis

### 2.1. Microvesicle Biogenesis

**Figure 1:**
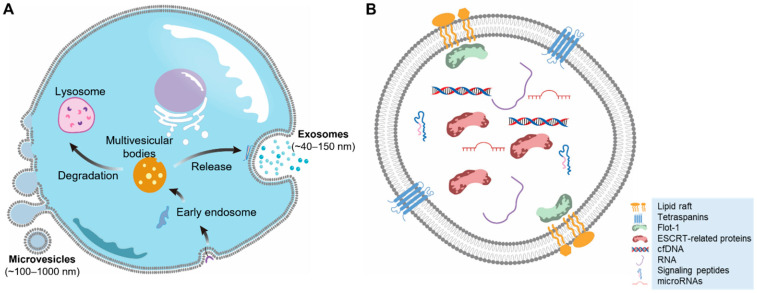
Extracellular vesicles (EV) subtypes based on size, biogenesis difference, and composition. (**A**) The major types of EVs are microvesicles (MVs, diameter: ~100–1000 nm) and exosomes (diameter: ~40–150 nm). The biogenesis of MVs and exosomes is generally different with certain shared pathways. (**B**) General EV compositions and cargos include peptides, lipids, proteins, genetic materials, and metabolites. MicroRNAs (miRs) are the major cargo in EVs and exhibit extensive functions in vitro and in vivo.

Depending on the cell type, culture conditions, and isolation strategies, a mixture of exosome and MVs can always co-exist, though the percentage may vary. This heterogeneity is because MVs and exosomes share similar characteristics at a certain range of sizes (Figure 1), which makes isolation and purification difficult. Based on size characteristics, MVs vary from 100–500 nm (ectosome), but can reach up to 1000 nm (which are referred to as oncosomes and are identified in highly invasive cancer cells) [4,5].

Both MVs and exosomes are distinctive populations compared to apoptotic bodies secreted through cell apoptosis [6]. Although sharing the similar membrane budding release, the biogenesis of MVs is quite different from that of exosomes. MVs undergo fission and directly bud outward at the plasma membrane (PM) of cells. However, the heterogeneity of MVs (with a wide range of sizes from 100–1000 nm) suggests that multiple mechanisms could be involved during membrane shedding, such as phospholipid site alteration or PM blebs. These alterations and blebs keep expanding to generate outward membrane curvature for MV budding out from the lipid sites, where cytoskeleton reorganization is commonly observed [7,8,9,10,11].

**Figure 2:**
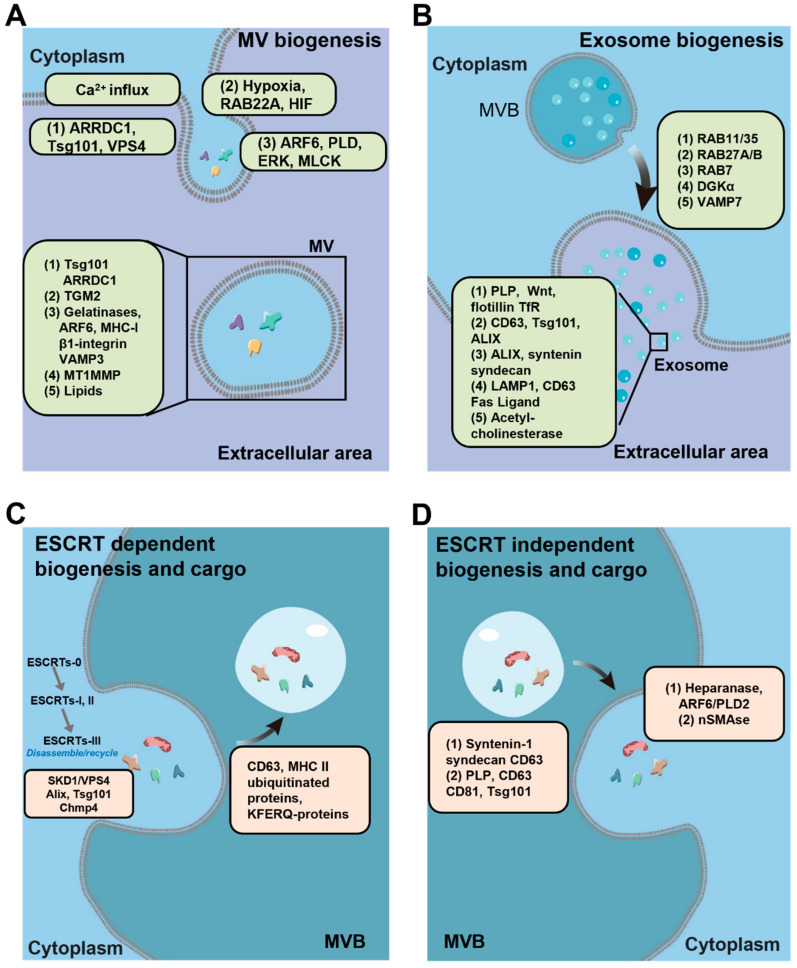
EV biogenesis pathways. (**A**) MV biogenesis. Plasma membrane rearranges at specific sites following Ca^2+^ influx, which recruits enzymes such as scramblase and floppase. ARRDC1 regulates ESCRT proteins TSG101 in an ATP-required manner (VPS4) to release MVs. Other modifications such as hypoxia-induced factors (HIF) and ARF6-stimulated PLD-ERK activation of myosin light chain kinase (MLCK) can also induce MV biogenesis. Associated proteins and molecules can be found in secreted MVs. (**B**) Exosome biogenesis and associated proteins promote MVB fusion to PM and regulate specific cargo packaging. (**C**) ESCRT-dependent exosome biogenesis. ESCRTs act in a stepwise manner to generate exosomes in cytosol and regulate protein cargo packaging. (**D**) ESCRT-independent pathways and associated proteins.

The cytoskeleton rearrangement results in the generation of the budding neck and eventually rupture of the membrane to release MVs following Ca^2+^ level changes (Figure 2A) [12]. Several substances on the cell membrane have been identified for MV biogenesis: (1) phosphatidylserine—the most abundant anionic phospholipid in the cell membrane—can move from the inner to the outer leaflet of the PM via enzymatic reactions of flippases (ATP-driven); (2) floppases, which generate uneven force for membrane bending; and (3) ATP-independent scramblases, which redistribute phosphatidylserine and stabilize membrane rigidity [7,11,12]. In another case, acid sphingomyelinase (a-SMase), a lipid metabolic enzyme, mediates the P2X7 (an ATP receptor, generally found to be highly expressed in immune cells)-dependent release of large vesicles in glial cells (microglia and astrocytes), as the activation of P2X7 induces the translocation of a-SMase from lysosomes to the PM outer leaflet and further alters PM physical conditions [13,14]. This translocation of a-SMase can be achieved by stimulation of other receptors to enhance MV biogenesis [15]. In addition, MVs are mostly enriched with lipid rafts (containing cholesterol), which are associated with the proteins responsible for cytoskeleton reorganization (e.g., calpain, which is regulated by extracellular Ca^2+^, and ADP-ribosylation factor 6 (Arf-6), which is synchronized with the RhoA and ROCK signaling pathways for oncosome release) [16,17].

Another mechanism for MV biogenesis involves endosomal sorting complexes required for transport (ESCRT) proteins and cytoskeleton interactions. Arrestin domain-containing protein 1 (ARRDC1), which is an accessory protein of tetrapeptide PSAP motifs, acts as an adaptor that binds the PM to produce MVs. TSG101 relocates from the endosomes to the PM and binds to ARRDC1 through tetrapeptide PSAP motifs and promotes the release of MVs. Thus, these MVs contain TSG101, ARRDC1, and other late exosomal markers such as CD63 and LAMP1 [18]. The ESCRT-dependent biogenesis of MVs requires ATPases, such as vacuolar protein-sorting-associated protein 4 (VPS4), to enable the final pinch-off of the membrane and release of MVs [19,20,21]. In summary, MV subpopulations with unique cargo and functions may exist and different cell types and culture conditions can further complicate the heterogeneity of MVs. The proteins involved in MV biogenesis are summarized in Table 1.

### 2.2. Exosome Biogenesis

Exosomes are considered as small EVs (Figure 1A). Cargo analysis reveals the diversity of their contents, which include membrane receptors, soluble proteins, lipids, RNAs, metabolites, and organelles, leading to functional variance in recipient cells (Figure 1B). As shown in Figure 1A, exosomes originate at the internal endosomal membranes of multivesicular bodies (MVBs). Initiated by the endo-lyososomal pathway or endocytosis, early endosomes are generated from the PM, they bud into cytosol for maturation, and then are packed with MVBs or multivesicular endosomes (MVEs) regulated in a specific protein-cargo manner (Figure 2B). The inward budding of the endosomal membrane in MVBs/MVEs results in the accumulation of intraluminal vesicles (ILVs, precursors of exosome). The accumulated ILVs are released into the extracellular environment upon the fusion of MVBs/MVEs with the PM, now termed as exosomes [27,28]. Although it is difficult to dissect exosome biogenesis into clearly separated pathways, the current knowledge classifies exosome biogenesis into two categories: ESCRT-dependent and ESCRT-independent pathways.

#### 2.2.1. ESCRT-Dependent Pathways

ESCRT proteins represent the major machinery that regulates both MV and exosome biogenesis and can be grouped into five distinct complexes: ESCRTs -0, -I, -II, -III, and Vps4 [19,29], together with the accessory protein ALIX (Table 2) [30]. As reviewed above, TSG101 participates in the abscission of the vesicle bud from the PM to promote MV secretion, which is identified as an ESCRT-I complex process [23]. 

For exosome formation, all the ESCRT complexes relocate at the endosomal membrane of late endosomes and function in a stepwise manner to drive cargo sorting/packaging, vesicle budding, and fission (Figure 2C). 

The ESCRTs-0 complex initiates the process at the endosomal membrane after being recruited by phosphatidylinositol 3-phosphate. It recognizes and sequesters ubiquitylated proteins such as clathrin, ubiquitin, and other activated growth factors receptors [45]. Two major subunits of ESCRTs-0, HRS, and STAM1/2, bind ubiquitinated cargos and may be responsible for different subpopulations of exosomes [30]. Depletions of HRS and STAM1/2 have less effect on early endosomes but enlarge the MVBs, partially explaining the decrease during the production of small-size EVs [46]. Clearly, different subunits of ESCRTs-0 regulate specific exosome subpopulations. 

The ubiquitinated proteins and receptors are then passed along to ESCRTs-I and -II complexes, which provide ubiquitin-interaction domains to sort ubiquitinated cargos. ESCRTs-I and -II are mainly responsible for membrane deformation to accumulate ILVs [47]. Depletion of ESCRT-I protein TSG101 leads to an altered exosome protein profile (enriched CD63 and MHC-II negative vesicles) [30]. In addition, depletion of TSG101 also alters early endosome morphology by causing vacuolar domain alteration and further inhibits MVB formation [46]. Interestingly, overexpressed TSG101, however, also inhibited EV production, especially in an exosome size range of 30–100 nm [48].

Finally, ESCRTs-I and -II recruit ESCRTs-III monomers in the cytosol and reassemble them in active complexes (charged multivesicular body proteins, i.e., CHMPs) at the endosomal membrane via interactions with Alix and ESCRTs-I complexes. The activated complexes form filaments/spirals that drive the final step of ILV biogenesis, including cargo crowding, membrane deformation/budding, neck tightening, and scission of ILVs [49,50]. Then, ESCRTs-III spirals disassemble into small filaments and monomers, which are recycled in cytosol. Knock-out of ESCRTs-III protein CHMP1 resulted in reduced formation of MVBs but with an enlarged morphology [51]. VPS4/SKD1 disassembles and recycles ESCRTs-III complexes, while deficient ATPase caused enlarged MVBs and the accumulation of nonreleasable particles [52,53].

ESCRTs also interact with accessory proteins, namely, Syntenin, Syndecan, and Alix, for exosome biogenesis. Syntenin, as a cytoplasmic adapter protein, is found to interact directly with Alix via protein motifs [54]. Syntenin also binds to Syndecans at their cytosolic tails, and Alix connects the Syndecan–Syntenin complex to ESCRTs-III to eventually form ILVs. Direct evidence of this process can be observed in the fact that exosomes derived from MCF-7 cells perturbated with heparanase (the only mammalian enzyme that cleaves heparan sulfate of oligomerized Syndecans) exhibit different Syntenin-1, Syndecan, and CD63 protein profiles [55]. Moreover, recent studies revealed that the formation of exosomes via Syntenin/Syndecan/Alix/ESCRT-III is regulated by Arf6 and its effectors, phospholipase D2 (PLD2), which is translocated from PM to MVB lumen and enriched on secreted exosomes [56,57]. Alix/ESCRTs-III interactions are considered as ESCRT-independent in some studies [36,40,41].

#### 2.2.2. ESCRT-Independent Pathways

Interestingly, even with the complete abolishment of ESCRT functions, a certain level of ILV formation and exosome secretion still remains (although with altered subpopulation), which indicates that there is an ESCRT-independent pathway for exosome biogenesis (Figure 2D) [58,59]. For example, inhibition of neutral sphingomyelinase 2 (nSMase2) leads to the decreased production of sphingolipid ceramide-enriched exosome [60]. Other lipid interactions, such as sphingosine1-phosphate with metabolized ceramide, promote exosome release and cholesterol redistribution, thus influence cargo packaging [61,62,63]. The tetraspanin family, a series of proteins, can regulate the dynamic membrane domains, and influence exosome biogenesis independent of ESCRTs. For instance, CD63 is particularly enriched in exosomes and regulates endosomal cargo targeting and sorting, as well as protein trafficking and packing into exosomes [64,65,66,67,68]. Other tetraspanin proteins, such as CD9, CD81, CD82, Tspan6, and Tspan8, exhibit different mechanisms at different steps of exosome formation [69,70,71,72,73]. In summary, both the ESCRT-dependent and ESCRT-independent pathways are equally important for exosome biogenesis and operate simultaneously. Future investigations of EV biogenesis are required in order to fine-tune these biogenesis pathways for EV engineering.

## 3. Exo-miRNA Loading and Sorting in EVs

Typically, a complex cargo profile can be found in EVs regardless of cell type and culture conditions. On the other hand, the cargo profile varies dynamically depending on the cellular microenvironment and tissue origins. Therefore, understanding the cargo sorting mechanism is critical for engineering therapeutic EVs by manipulating culture conditions. As mentioned above, ESCRT complex is responsible for EV biogenesis and cargo recognition by providing distinct ubiquitin-binding motifs. For example, the ESCRTs-0 complex, with both HRS and STAM1/2 subunits, can bind to ubiquitin by its ubiquitin-interacting motif and recognize polyubiquitinated proteins [74,75]. Moreover, ESCRTs-0 also binds to the clathrin heavy chain via its clathrin box motif [45,76]. Similarly, ESCRTs-I and -II also provide ubiquitin-binding domains, which are not identified in ESCRTs-III [47,77]. Interestingly, EV secretion with functional cargos still occurs with the complete deletion of ESCRTs, implying other components, such as a lipid raft and ceramide, may also regulate the protein sorting process independent of ESCRTs [30,58,78,79,80,81].

Another important set of cargos in EVs are nucleic acids including DNAs, mRNAs, and miRs. It is highly possible that the cytoplasmic DNA fragments generated by the nucleus or mitochondria (due to DNA damage repair or DNA metabolism) are directly encapsulated into EVs [82,83,84]. However, other studies indicated lower levels of DNA cargo in nontransformed cell lines or in the circulatory system of healthy people compared to cancer cell lines and cancer patient cells [85,86]. Besides the extrusion of damaged genetic materials to maintain cellular homeostasis, exosomal DNAs also contribute to immunomodulatory functions in cancer therapy and could act as liquid biopsy markers for diagnosis [85,87,88,89,90]. However, little is known concerning their extensive functions in stem cell therapy and tissue development.

MiRs (with lengths of 19–24 nt) play important roles in inhibiting the expressions of target protein-coding genes and fit well with the function of EVs. MiRs were reported to make up the highest proportion of nucleic acids enriched in EV cargo along with other RNAs including mRNA, ribosomal RNA, long noncoding RNAs, and circular RNAs [91,92,93,94,95]. Interestingly, deep sequencing revealed that EVs generally had a distinguished miR profile compared to their parent cells, implying the regulated sorting rather than random packaging of miRs in EVs [75,77,80,81]. 

**Figure 3:**
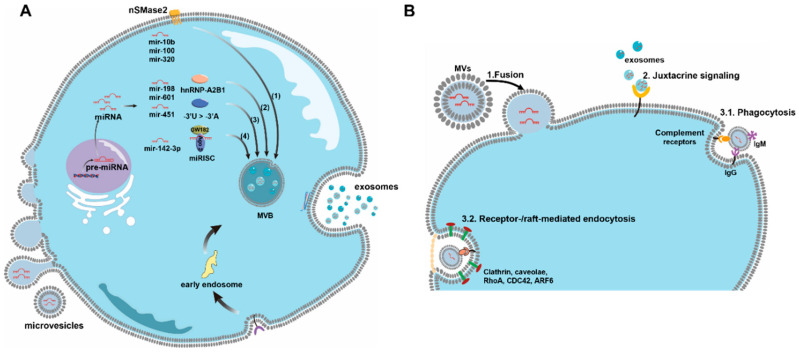
Exosomal cargo sorting and EV uptake by recipient cells. (**A**) MicroRNAs are sorted through different mechanisms/pathways into MVs and exosomes. Nucleus-released miRs in cytosol can be directly packed into MVs. For the exosome (MVB in cytosol), (1) nSMase2 regulates ceramide biosynthesis for selective miR sorting; (2) hnRNP proteins bind to specific miR motifs for sorting; (3) the modification of noncoding RNAs regulates 3′-end adenylated miR isoforms; (4) GW182 and Ago2 co-localized with MVB accumulate in the miR-induced silencing complex (miRISC). (**B**) Potential EV uptake mechanisms by recipient cells. MVs can directly fuse with the plasma membrane (PM) to deliver exosomal cargo. EVs can also bind to specific sites on the PM to exert juxtacrine signaling to activate intracellular pathways. Alternatively, EVs can be phagocytosed or endocytosed via specific receptor mediation on recipient cells.

Obviously, cellular/cytosol abundance of miRs is associated with their sorting into EVs [96], although the mechanism is still not well understood. Based on the current knowledge and evidence, several pathways have been proposed as the mechanisms for miR sorting into EVs (Figure 3A):

(1) The modulation of lipid biosynthesis could influence both EV biogenesis and miR sorting. As reviewed above, ceramide is critical for exosome release and protein targeting in EVs. Disrupting ceramide biosynthesis by the inhibition of nSMase-2 leads to a reduction in miR-16 and miR-146a levels in EVs [80,97]. The modulation of nSMase-2 has been established for perturbing the sorting of other miRs in EVs, such as miR-10b, miR-100, and miR-320 [64,84,85]. However, alteration of exosomal miRs by modulating lipids is risky since the integrity of EVs can be significantly impacted as lipids are crucial in EV biogenesis.

(2) MiR sorting into EVs is also dependent on the specific sequence/motifs interactions with binding proteins. In Jurkat cell-secreted exosomes, over 70% of the exo-miRs have a GGAG motif (or an extra seed sequence) in the 3′-portion of miR, which is a binding site for sumoylated heterogeneous nuclear ribonucleoprotein (hnRNP), or hnRNP-A2B1 specifically [98]. Other types of hnRNP proteins can bind to specific motifs of miRs and then regulate miR sorting into EVs [98,99]. In addition, RNA-binding proteins, such as Y-box protein 1 (YBP-1), also regulate miR sorting into EVs, although the binding motifs are not identified [100,101].

(3) Similar to the specific binding motifs, the 3′-end nontemplate sequence in miRs also plays certain roles in cargo sorting into EVs. For example, 3′-end uridylated miR isoforms are mainly expressed in exosomes, while 3′-end adenylated miR isoforms are relatively enriched in B cells [102]. Although this post-transcriptional modification of noncoding RNA seems to drive cytoplasmic Y RNA sorting, more evidence is required to elucidate its general role in sorting other miRs. 

(4) MiR sorting could also be mediated by miR-induced silencing complex (miRISC). The major components of miRISC found in monocytes are miRs, miR-repressible mRNAs, and GW bodies (GW182 and Ago2) co-localized with MVBs, all of which were determined by immunofluorescent staining of RISC-MVB markers [103,104]. Studies also utilized the inhibition of ESCRT to block the turnover of MVBs into lysosomes, which leads to the accumulation of miRISC. On the other hand, disrupting MVB formation causes the loss of miRISC and relieves miR-mediated gene silencing [103]. These are the first pieces of evidence showing that miRISC and MVBs are both physically and functionally associated. Further studies indicate that knockout of Ago2 could eliminate or decrease the expression of certain exosomal miRs, such as miR-451, miR-150, and miR-142-3p in HEK293T cells [91]. Moreover, Ago2 can sometimes be expressed in exosomes [105]. In addition, elevating the cellular levels of miR-repressible mRNAs also contributes to the enrichment of target miRs in MVBs and facilitates miR sorting [96]. This evidence may imply that miRISC is involved in miR sorting into EVs. However, to better modulate EV content and the miR profile via miRISC, more investigations are needed.

## 4. Mechanism for EV Uptake by Recipient Cells and Exosomal miRNA Functions

Cell–cell communication represents the most important role of EVs in cellular events and tissue development. The heterogeneity of cargo, surface components, and sizes influence the uptake of EVs by recipient cells. EVs can interfere with cellular pathways and behavior by binding to the target cell surface without delivering any cargo. One widely observed example is the activation of T lymphocytes [106]. During the immune response, B lymphocyte-secreted EVs with major histocompatibility complex (MHC) class II-enriched compartments can directly activate antigen-specific MHC class II-restricted T cells without delivery of cargo [107]. Similarly, dendritic cells also secrete EVs with MHC-peptide complexes for the activation of T lymphocytes, although different EV subtypes exert different capacity for activation [108]. The mechanism of this direct binding has encouraged research on manufacturing EV mimics with functional surface markers that regulate immune responses. However, the main interest in the functionality of EVs is the delivery of cargo to the recipient/target cells. Thus, understanding the uptake of EVs and the fate of the delivered exosomal cargo is critical for the future design of EV-based therapies.

In general, three major interactions exist between EVs and recipient cells and these interactions are highly dependent on the specificity of EVs (Figure 3B). For instance, directly binding and docking on the recipient cell PM is likely to be regulated by Tetraspanin proteins, adhesion molecules (e.g., integrins, ICAMs, and lectins), lipids, proteoglycan, and the extracellular matrix [70,109,110,111,112]. After binding, EVs can stay at the membrane without delivering cargo as discussed above, where EVs act as ligands or intracellular signal mediators to regulate recipient cell behaviors [113]. This juxtracrine fashion of interaction eventually ends up with the release of binding EVs instead of internalization. In other cases, EVs enter the recipient cells by phagocytosis, macropinocytosis, or receptor-mediated endocytosis, where endosomes are the destination for cargo delivery. Alternatively, EVs can also enter the recipient cells by fusion with the cytoplasm membrane to directly release the intraluminal cargo inside recipient cells [114,115,116,117,118,119,120,121,122]. The ultimate fate of EVs is degradation by lysosomes to recycle the compartment for re-secretion [123]. The internal trafficking of EVs also requires cellular components from recipient cells, although the specific mechanism is unknown.

## 5. Engineering and Therapeutic Strategies with Exosomal miRs in Regenerative Medicine

### 5.1. Exo-miR from Mesenchymal Stem Cells (MSCs) in Bone-Associated Regeneration

**Figure 4:**
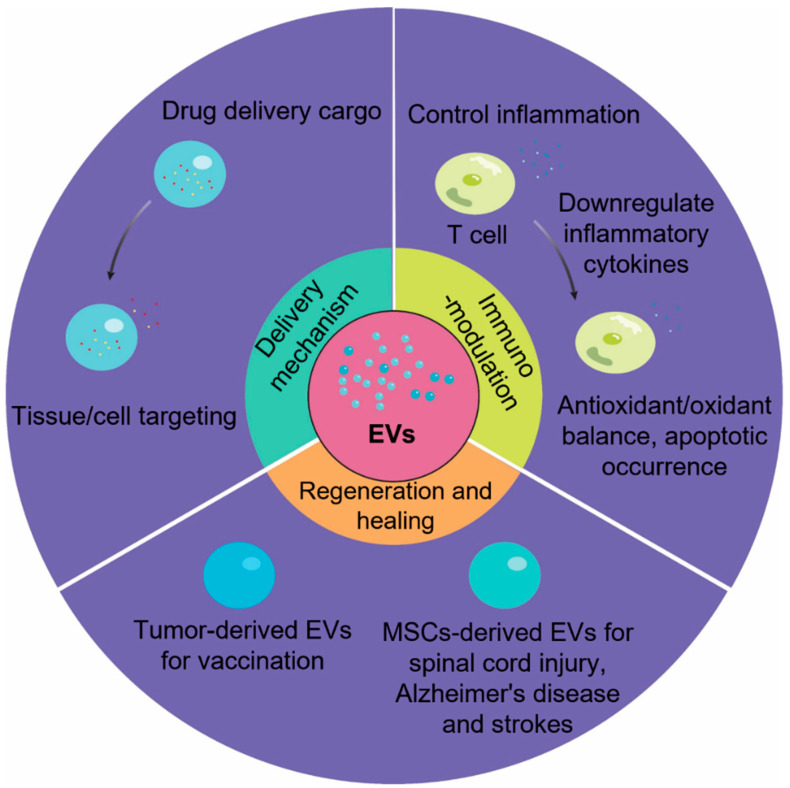
Potential engineering strategies in EV therapeutics. Based on their bioactivity and biostructure, EVs or EV mimics have been engineered to deliver therapeutic molecules. The immunomodulatory potentials of EVs have been applied for the relief of inflammatory sites or tissue damage. EVs from specific cell sources can be directly used as a cell-free therapy or for vaccination purposes. With the understanding of EV biogenesis and cargo sorting, the desired size subpopulation and cargo profile can be engineered via altering the cellular microenvironment of in vitro cultures.

After being incorporated by recipient cells, EV cargos such as exo-miRNAs exhibit extensive regulations on various cellular behaviors in different tissue and potential therapeutic efficacy in disease models (Figure 4). For instance, mesenchymal stem cell (MSC)-derived exosomes have drawn major attention due to their broad therapeutic impacts in multiple diseases and the exo-miRs of MSC-derived EVs were proposed as the major bioactive compartments for those promising results. In bone-associated diseases, exosomal miR-150-3p promotes osteoblast proliferation and differentiation in osteoporosis and establishes potential targets in osteoporosis treatment [124]. Another study points out that EVs from MSCs during different osteogenic stages induced bone formation differently, due to the fact that their miR profile (such as miR-31, -144, and -221 as negative regulators) was sequentially changed from the expansion stage to osteogenic differentiation stage [125]. For cartilage regeneration, exosomal miR-92a-3p could regulate chondrogenesis and extend cartilage development and homeostasis by targeting WNT5A for osteoarthritis treatment [126]. MiR-148a and -29b enriched in Wharton’s jelly mesenchymal stem cell-derived (WJMSC) EVs promote cartilage repair by regulating lineage commitment towards chondrogenesis instead of hypertrophic phenotype [127]. These studies suggest that regeneration may come from MSC paracrine effect rather than direct osteogenesis or chondrogenesis.

### 5.2. Exo-miR from MSCs in Cancer Treatment

In cancer models, MSC EVs also demonstrated potential regulations. Specifically, exosomal miR-139-5p inhibits bladder tumorigenesis by targeting the polycomb repressor complex 1 and miR-15a delays carcinoma progression by inhibiting spalt-like transcription factor 4 [128,129]. EVs enriched with miR-497 showed effective inhibition of tumor growth and angiogenesis [130]. On the other hand, exo-miRs (e.g., miR-21, miR-155, miR-146a, miR-148a, and miR-494) derived from tumor cells or activated macrophages can promote angiogenesis and immune escape to facilitate cancer metastasis in new studies [131,132,133,134]. Thus, exo-miRs can generally act as biomarkers in cancer diagnosis and prognosis. This complex functional diversity of exo-miRs suggests the needs for thorough evaluations of specific miRs in a case-by-case manner before defining the exo-miR profile for cancer treatments. 

### 5.3. Exo-miR in Alzheimer’s Disease Pathology and Treatment

Another potential application of MSC-derived EVs is for Alzheimer’s disease (AD). For instance, WJMSCs produced exosomes enriched with miR-29a, which specifically target histone deacetylase 4 (HDAC4, an elevated marker in AD patients). A reduction in Aβ expression and improved cognitive recovery were observed after MSC EVs treatment, and a significant decrease in nuclear HDAC4, with a certain amount of HDAC4-related gene fluctuation [135,136]. Similarly, miR-29-enriched EVs also reduced the toxic effects of Amyloid β (Aβ) peptide and partially recovered cognitive impairment in rat AD models [137]. In another study, exosomal miR-21 was found to be enriched in EVs from hypoxia-preconditioned MSCs and ameliorated cognitive decline by regulating neuroinflammation and synaptic damage [138]. Interestingly, MSCs can be manipulated by altering in vitro culture conditions (e.g., hypoxia or 3D aggregation) and certain cellular changes will be captured in their secreted EVs [138,139]. Injection of 3D hMSC-derived EVs with a spectrum of upregulated miRs (such as miR-21, miR-22, and miR-1246) effectively prevented cognitive declines in AD mice [140]. Moreover, miR-21-5p from human urine-derived stem cell-derived EVs attenuated Rett syndrome, an early cognitive loss and neurologic deterioration disease, through the inhibition of Eph receptor A4 (Epha4) and its downstream signaling TEX [141]. Pathologically, cerebral EVs have been proved to contain amyloid precursor protein (APP) and C-terminal fragments (CTT), which all contribute to Aβ and tau protein accumulation [117]. In addition, EVs also carry neurotoxins and inflammation molecules, which further facilitate AD progression. Understanding the biological roles of EVs in disease pathology provides the possibility of designing EV-based vaccinations with exo-miRs, such as cell-free cancer immunotherapy (Figure 4) [142,143]. These studies indicate the existing connections between AD progression and exo-miRs, although the exact mechanisms need to be elucidated to achieve optimized miR cargo for AD therapy. 

### 5.4. Exo-miR in Spinal Cord Injury and Treatment

In spinal cord injury (SCI), neurological repairs require the regulation of miRs for neurogenesis, neural differentiation, and neural tube formation. A set of circulating exo-miRs have been revealed to be upregulated (miR-9, -124a, -7, -125a/b, and -375) or downregulated (miR-291-3p, -183, -92, -200b/c, and -382-5p) during this process in rodent models [144,145]. Therapeutically, MSC-derived EVs containing miR-126, -133b, -21, or -199a-3p/145-5p have shown potential therapeutic benefits to neuron regeneration and immunomodulation to promote functional recovery, possibly due to the complex regulatory mechanisms on RhoA, STAT3, and the Pi3k/Pten/Akt axis, etc. [144,146,147,148,149]. Moreover, EVs derived from neurons, microglia, and oligodendrocytes also provide neuroprotective effects via miRs after SCI, namely, miR-21 [150,151], -124-3p [152], -9, and -19a [153]. With these potential cargo candidates, artificial EVs or EV-mimic particles can be optimized for SCI treatment [154].

### 5.5. Exo-miR from MSCs in Ischemic Diseases

For ischemic diseases, EVs and miRNAs also exhibited promising therapeutic efficacy. For instance, ischemic stroke caused by oxygen and nutrient deprivation leads to severe lesions and neurological damage. MSC EVs loaded with miR-138-5p (specifically targeting lipocalin) prevented further astrocyte damage by oxygen/glucose deprivation (OGD) after endocytosis and thus alleviated lesion damage in ischemic mice [155]. Activating transcription factor 3 (ATF3) was upregulated in a rodent stroke model and later identified as a target of miR-221-3p. MSC-derived EVs loaded with miR-221-3p could be recognized by neurons, reduced local inflammation, and eased cellular death caused by OGD, via suppression of ATF3 expression in neurons [156]. Similarly, exosomal miR-146a-5p also modulated neuroinflammation via microglia in ischemic stroke, and the proposed target is the IRAK/TRAF6 signaling pathway. MiR-133b was found to be transferred by MSC EVs, promoted neural plasticity, and enhanced neurite outgrowth in MSC-based stroke treatment [157,158]. Those studies demonstrate the important role of EVs and miRs in neural tissue regeneration. In ischemic cardiomyocyte injury, one study showed that MSC EVs treatment could inhibit cardiomyocyte apoptosis caused by hypoxia/reoxygenation. Exosomal miR-486-5p targeted Pten in this process, thus activating the Pi3k/Akt survival pathway and extending the protective effect in mice I/R (ischemia/reperfusion)-injured myocardium [159]. Another study revealed that miR-126 improved cell survival in neonatal rat ventricular cardiomyocytes cultured under H_2_O_2_ and CoCl_2_. MiR-126 binds to ERRFI1 protein to exhibit antioxidative effects and restore mitochondrial fitness, thus improving resistance to I/R in vivo [160]. MiR-182 in mouse bone marrow-derived MSC EVs was also proposed to regulate macrophage polarization by downregulating TLR4 and NF-κB, thus attenuating myocardial injury. Both miRNA-133a and miRNA-141 from MSC exosomes exhibited myocardial protection through the downregulation (via suppressing mastermind-like 1) and upregulation (via PTEN) of β-catenin, respectively [161,162]. These studies have aroused broad interests in the complicated miR profile and functions in EVs, which may lead to many applications in therapeutic engineering, although further validation is required. 

### 5.6. Exo-miR Detection and EV Biomanufacturing

Recently, researchers have pushed forward the detection sensitivity and variety of exo-miRs with novel engineering strategies, such as microfluidic chips, to enrich EVs and the in situ detection of exo-miRs with catalyzed hairpin assembly [163]; nanochannel biosensors coated with functional peptide nucleic acids to achieve charge alteration for EV capture and high resolution detection [164]; and self-assembled tetrahedral DNA nanolabel-based electrochemical sensors to selectively detect exo-miRs [165]. A new subpopulation of EVs (e.g., exomere, <50 nm) was even identified recently with asymmetric-flow field-flow fractionation (AF4), which further elucidated the dynamic composition of proteins, small RNAs, and lipids in EVs [166,167]. Besides the popular delivery system with EV-inspired liposome to load doxorubicin and kartogenin or other therapeutic drugs [168], fully synthetic/engineered EVs with the precisely controlled composition of miR cargo and the specific rations between miRs (i.e., stoichiometry) were engineered for wound healing and keratinocyte function, achieving similar effects compared to natural EVs [169]. With increased knowledge of exo-miRs and other cargo profiles in EVs, further modified/engineered EVs with therapeutic biomolecules are a promising alternative for next generation targeting therapies in tissue engineering and regenerative medicine. Engineering EVs for biomanufacturing is also critical for translational applications. While the cellular source of EVs is potentially case/disease dependent, the general implication for EV production is to scale-up and boost yield. Recent studies demonstrated that traditional suspension and microcarrier-based bioreactors are able to support the scale-up of cells and EVs [170]. When cultured in larger scale vertical-wheel bioreactors (0.5 Liter vs. 0.1 Liter), hMSC-EV production was well maintained or slightly boosted with similar proteomics and metabolomics, despite the shear stress impact on cultured cells. A similar bioprocessing approach has been tested in a hollow fiber bioreactor [171]. Moreover, a nonadherent culture of MSCs under a WAVE bioreactor also boosted EV yield threefold in a recent study [139]. The 3D aggregation process of hMSCs significantly altered EV production and the cargo profile compared to a 2D adherent culture, exerting different cargo profiles and advanced functions in immunomodulation and rejuvenation. In addition to the 3D aggregation of hMSCs for EV production [172,173,174], the application of a wave motion bioreactor enables the closed-system scale-up of EV bioprocessing and biomanufacturing. Interestingly, these studies also indicate the potential engineering strategy of EVs by manipulating the in vitro microenvironment. 

## 6. Summary

The EV world is complex and fascinating. The complexity of EV heterogeneity, biogenesis, and important cargo miRs encourages us to further explore the biology in order to further understand the nature of vesicles. This review summarizes the basic concepts and current knowledge on exo-miRs and their sorting mechanisms in the parent cells for designing and engineering therapeutic cargos in EV-based therapies. Applications and preclinical studies indicate the potential therapeutic effects of exo-miRs in multiple disease models. Continuous studies are needed as our knowledge evolves in the EV field with advanced technologies and experimental strategies. As exo-miRs have been shown to participate in disease progression in ischemia and neurodegeneration, they are promising for potential cell-free therapies using fully synthetic EVs with defined miR and protein cargo neurological restoration. 

## Figures and Tables

**Table 1:** Proteins involved in MV biogenesis.

Proteins	Location	Functions	Ref
ARF1	Golgi apparatus and shedding microvesicles	Regulation of matrix degradation by directly acting on the structures associated with invasiveness—invadopodia maturation and the shedding of membrane-derived microvesicles	[10]
ARF6	Plasma membrane, cytosol, and endosomal membranes	Regulating the actomyosin-based membrane abscission mechanism to control the shedding of microvesicle in tumor cells	[7]
Rab22a	Nonclathrin-derived Endosomes, budding microvesicles	Increasing microvesicle shedding in human breast cancer under hypoxic conditions and knockdown of RAB22A impairs breast cancer metastasis	[22]
RhoA	Membrane and cytosol	Involved in microvesicle biogenesis through regulation of myosin light chain phosphatase. required for microvesicle shedding	[11]
ARRDC1	Plasma membrane	ARRDC1-mediated relocalization of TSG101 may alter endosomal trafficking and sorting and signal transduction by receptors subjected to endosomal sorting mechanisms	[23]
DIAPH3	Plasma membrane, Microtubules/microvilli	DIAPH3 silencing also promotes shedding of extracellular vesicles (EV) containing bioactive cargo and increases proliferation of recipient tumor cells, and suppresses proliferation of human macrophages and peripheral blood mononuclear cells	[24,25]
Myosin-1a	Plasma membrane	Enterocyte microvilli containing Myosin-1a are active vesicle-generating organelles	[26]

**Table 2:** Proteins involved in ESCRT pathways.

Complex		Location	Cargo Sorting	Functions	Ref
*ESCRTs-0*	HRS	VHS, FYVE, P(S/T)XP, GAT domain and coiled-coil core, clathrin-binding	Binding to/clustering with ubiquitinated cargo for delivery into MVBs, and recruits clathrin, ubiquitin ligases, and deubiquitinating enzymes, and almost certainly has other functions as well	Clustering of Ub cargo, MVB biogenesis	[31]
	STAM1/2	VHS, UIM, SH3, GAT domain and coiled-coil core			[32]
*ESCRTs-I*	TSG101	UEV, PRD, stalk, headpiece	Binding ubiquitinated cargo, ESCRT-0, ESCRT1, BRO1 and viral proteins	Membrane budding, MVB biogenesis, viral budding, replication and cytolinesis	[33,34]
	HVPS28	headpiece, Vps28 CTD	ESCRT-0, ESCRT1, BRO1 and viral proteins		[35]
	VPS37	basic helix, stalk, headpiece	Membrane binding		[36]
	hMVB12	stalk, headpiece (“UMA domain”), MAPB	N/A		[37]
*ESCRTs-II*	EAP20/VPS25	Winged-helix	Binding ubiquitinated cargo, binding to human ESCRT-I	The essential partner of ESCRT-I in MVB biogenesis and budding formation, membrane budding	[38]
	EAP30/VPS22	basic helix, Winged-helix	Forming nearly equivalent interactions with the two Vps25 molecules		[39]
	EAP45/VPS36	Winged-helix, GLUE,	Binding PI containing membranes, ubiquitinated cargo and ESCRT-1-i		[40]
*ESCRTs-III*	CHMP2/VPS2	MIM1	Recruits VPS4, initiates ESCRT disassembly	Membrane scission	[41]
	CHMP3/VPS24	weak MIM1	Caps Snf7 polymer, recruits VPS2		[41]
	CHMP4/SNF7	weak MIM2	Main driver of membrane scission, bind Bro1		[42]
	CHMP6/VPS20	MIM2	Binding ESCRT-II and Doa4, acts as nucleator of Snf7 polymer		[41]
*VPS4*	SKD1/VPS4	MIT, AAA	AAA ATPase disassembles ESCRT-III, active function in MVB membrane scission	Vps4 solubilizes ESCRT-III subunits at the cost of ATP hydrolysis. LIP5 binds to Vps4 and promotes its oligomerization, activity, and ESCRT-III binding	[43]
	LIP5	MIT	Binding vps4 to promote ESCRT-III recycling		[44]

## Data Availability

Not applicable.
